# Spatial separation of photo-generated electron-hole pairs in BiOBr/BiOI bilayer to facilitate water splitting

**DOI:** 10.1038/srep32764

**Published:** 2016-09-02

**Authors:** Zhen-Kun Tang, Wen-Jin Yin, Le Zhang, Bo Wen, Deng-Yu Zhang, Li-Min Liu, Woon-Ming Lau

**Affiliations:** 1 Beijing Computational Science Research Center, Beijing 100084, China; 2 College of Physics and Electronics Engineering, Hengyang Normal University, Hengyang 421008, China; 3 Center for Green Innovation, School of Mathematics and Physics, University of Science & Technology Beijing, Beijing 100083, China

## Abstract

The electronic structures and photocatalytic properties of bismuth oxyhalide bilayers (BiOX1/BiOX2, X1 and X2 are Cl, Br, I) are studied by density functional theory. Briefly, their compositionally tunable bandgaps range from 1.85 to 3.41 eV, suitable for sun-light absorption, and all bilayers have band-alignments good for photocatalytic water-splitting. Among them, heterogeneous BiOBr/BiOI bilayer is the best as it has the smallest bandgap. More importantly, photo-excitation of BiOBr/BiOI leads to electron supply to the conduction band minimum with localized states belonging mainly to bismuth of BiOBr where the H^+^/H_2_ half-reaction of water-splitting can be sustained. Meanwhile, holes generated by such photo-excitation are mainly derived from the iodine states of BiOI in the valence band maximum; thus, the O_2_/H_2_O half-reaction of water splitting is facilitated on BiOI. Detailed band-structure analysis also indicates that this intriguing spatial separation of photo-generated electron-hole pairs and the two half-reactions of water splitting are good for a wide photo-excitation spectrum from 2–5 eV; as such, BiOBr/BiOI bilayer can be an efficient photocatalyst for water-splitting, particularly with further optimization of its optical absorptivity.

Photocatalytic water splitting has attracted worldwide attention for its potential in solving energy crises and environmental problems^1^. Although many catalysts do satisfy the requirement of band alignment with the reduction/oxidation chemical potentials for water-splitting in order to facilitate this important photocatalytic reaction, the efficiency of photocatalysis still remains too low to promote practical applications^2^. One critical deficiency arises from the fact that most of the current photocatalyst-candidates such as TiO_2_ have a wide band gap and are activated by ultraviolet but not visible sun-light. In addition, photo-generated electron-hole pairs in TiO_2_ and most photocatalysts tend to recombine instead of facilitating water-splitting. To overcome these deficiencies, many strategies have been developed in the past few decades^3^,^4^. Of these strategies, the approach of coupling two semiconductors to form a layer structure having an interfacial electric field is particularly promising because this both enhances the possibility of complying the band alignment requirements for water-splitting by band-structure engineering with two semiconductor constituents, and, at the same time, offers a means of separating photo-generated electron-hole pairs by engineering the interfacial electric field^5^,^6^,^7^,^8^,^9^.

In the development of novel semiconductor junctions, making heterostructures by coupling two compositionally-different layers of two-dimensional van der Waals (2D vdW) monolayers is certainly the most elegant approach^10^,^11^,^12^,^13^. Indeed, an exemplary heterostructure comprising a monolayer of graphene and a monolayer of hexagonal boron nitride (graphene/BN) has been demonstrated experimentally^14^,^15^,^16^. Similarly, many vdW heterostructures deriving from transition-metal dichalcogenides have been synthesized and characterized^17^,^18^,^19^. Theoretical studies of 2D vdW heterostructures have also predicted new physical properties such as heterojunction-induced spatial separation of photo-generated electron-hole pairs^20^, heterojunction-tuned band gaps^21^,^22^ and heterojunction-modulated nanometer-scale Moiré patterns^23^. These recent studies also suggest that the observed new physical properties are derived from the unique coupling of nanoscale confinement of electronic states and interfacial interactions in 2D vdW heterostructures. Further clarification of such underlying physics is critically important in developing new 2D vdW heterostructures, particularly in finding superior photocatalysts for efficient and practical water-splitting.

Among recent studies of 2D vdW monolayers, research on bismuth oxyhalides (BiOX, X = Cl, Br, I) has received particularly intensive attention in the field of photocatalytic water-splitting^24^,^25^,^26^,^27^,^28^,^29^,^30^. In this context, the family of BiOX is attractive because its members are semiconductors with bandgap easily tuned by changing X: 3.47 eV for BiOCl, 2.84 for BiOBr, and 1.87 eV for BiOI for bulk crystals^28^, and 3.79, 3.41, and 2.30 eV for their monolayer counterparts^29^. In addition, BiOX monolayers are known to possess band edge positions for water splitting. Among them, BiOI has higher photocatalytic activity than BiOCl and BiOBr under visible light irradiation, which is logical because the bandgap of BiOI is the smallest. The promising results of BiOX monolayers have also stimulated follow-up research on BiOX bilayers. Indeed, several experimental reports^24^,^25^,^26^,^27^,^28^ have already confirmed that like BiOX monolayers, BiOX bilayers are active in photocatalytic water-splitting.

Among all BiOX bilayers, heterolayers of BiOX_1_/BiOX_2_ with X_1_ and X_2_ being different halides are plausibly superior to homogeneous BiOX bilayers due to the possibility of heterojunction-induced separation of photo-generated electron-hole pairs. This prediction is supported by the experimental results of Cao, *et al*., who showed BiOBr/BiOI having photocatalytic water-splitting efficiency higher than the BiOX monolayer counterparts^30^. However, the enhancement mechanism leading to this observation is still not clear.

In this work, first-principles calculations were carried out to systematically examine the geometry, electronic structure, and optical properties of both homogeneous and heterogeneous BiOX bilayers. The properties of bandgap, band-structure, band-alignment in reference to water-splitting, optical properties, and photo-generated electron-hole separation were probed and compared.

## Results

**Figure 1:**
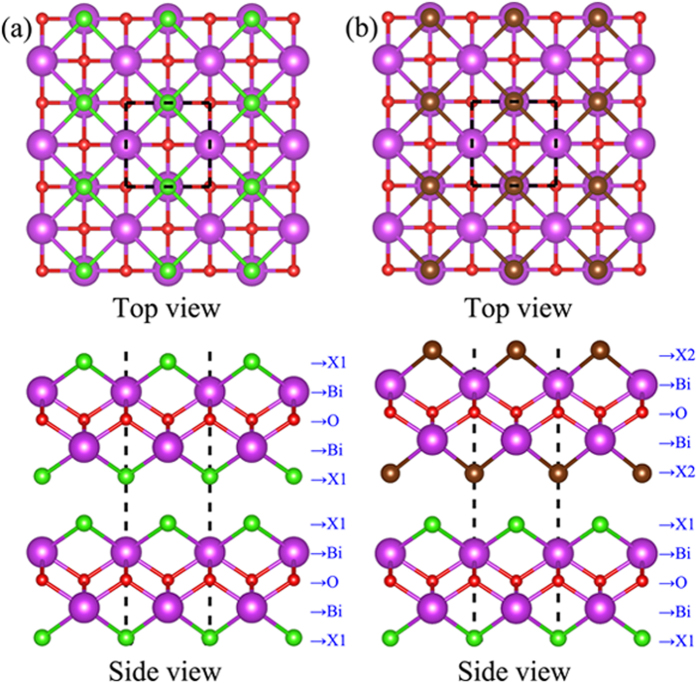
The relaxed atomic structures of the (**a**) homogeneous bilayer of BiOX/BiOX and (**b**) heterogeneous bilayer of BiOX1/BiOX2. The violet, red, green, and brown balls represent Bi, O, X1 (Cl), and X2 (Br) atoms, respectively.

Structural data are the very key parameters in understanding and using monolayers and bilayers of BiOX1/BiOX2. Our calculations show that monolayer BiOCl, BiOBr and BiOI have respective lattice constants of 3.87, 3.91 and 3.99 Å; this prediction agrees well with the known data in the literature^29^. Since these lattice constants do not differ much, heterogeneous bilayer structures can be formed readily with little interfacial strain. Our calculated structural results on homogeneous and heterogeneous bilayers are depicted in Fig. 1(a,b) and summarized in Table 1. In all bilayer cases, the two monolayer constituents couple themselves by aligning the X atoms of one BiOX constituent layer in proximity to the Bi atoms of the other BiOX layer. This coupling configuration is reasonable because the X atoms of each constituent layer are electronegative and thus tend to avoid each others and to get close to the Bi atoms which are eager to give up some their valence electrons. Interestingly, homogeneous bilayer formation consistently leads to very slight lattice dilation. For example, when two monolayers of BiOCl move towards each other from a well-separated condition to the fully relaxed form of a vdW bilayer, the lattice constant changes from 3.87 to 3.89 Å. Similarly, the respective lattice constants of vdW bilayer of BiOBr/BiOBr and BiOI/BiOI are 3.92 and 4.00 Å, with about 0.01 Å of lattice dilation. Such dilation probably eases the charge-repulsive forces between the X atoms of the constituent layers at the coupling interface. This also explains that the lattice dilation of BiOCl/BiOCl is larger than the other homogeneous bilayers, as Cl is more electronegative than Br and I. As for the heterogeneous bilayers, the respective lattice constants of BiOCl/BiOBr, BiOCl/BiOI, and BiOBr/BrOI are 3.90, 3.94, and 3.96 Å, respectively.

Direct and quantitative analyses of such coupling strengths are best conducted by measurements of bilayer formation energy. In this work, such formation energy data (expressed in eV per unit-cell) are calculated with the following definition:


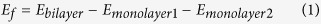


where *E*_*bilayer*_, *E*_*monolayer*1_ and *E*_*monolayer*2_ respectively represent the total energies of BiOX1/BiOX2, monolayer BiOX1, and monolayer BiOX2. The calculated formation energies are −0.38, −0.32, −0.32, −0.33, −0.27 and −0.29 eV, respectively for BiOCl/BiOCl, BiOBr/BiOBr, BiOI/BiOI, BiOCl/BiOBr, BiOCl/BiOI, and BiOBr/BiOI. The results thus confirm the formation of all these bilayers are thermodynamically favorable. The cohesive energy (expressed in J/m^2^) is the formation energy per unit area, which is experimental evaluation index for the interaction between layers. For a formation energy of −0.3 eV and a lattice constant of 0.4 nm, one can estimate the corresponding cohesive energy being about 1.0 J/m^2^. Recently, Zhou, *et al*.^31^,^32^ calculated that bilayers of graphene/graphene, BN/BN, and graphene/BN have respective cohesive energy of 0.56, 0.22, and 0.35 J/m^2^, with BN/BN being the weakest bilayer. Hence, all members of BiOX1/BiOX2 are considerably more stable and more strongly coupled than the case of graphene/graphene^32^. From another perspective, graphene/graphene stacking is easily formed experimentally and can even cause trouble in experimental research requiring monolayer graphene due to undesirable aggregation of graphene monolayers. In other words, BiOX1/BiOX2 bilayers are robust enough for practical applications.

Another important structural parameter of a vdW bilayer is the separation of the layer constituents, and this parameter figuratively reflects the coupling strength of the two layer constituents. The calculation results on layer separation are included in Table 1 and they clearly show that even for the weakest case of BiOI/BiOI, the layer separation of 3.01 Å is still shorter than the layer separation of the exemplary bilayer of graphene/graphene at 3.34 Å^33^ and the bilayer of BN/BN at 3.33 Å^31^. Hence the coupling strength of all BiOX1/BiOX2 combinations in this work should be higher than the case of pi-pi coupling of graphene/graphene and the case of weak ionic coupling of BN/BN.

**Figure 2:**
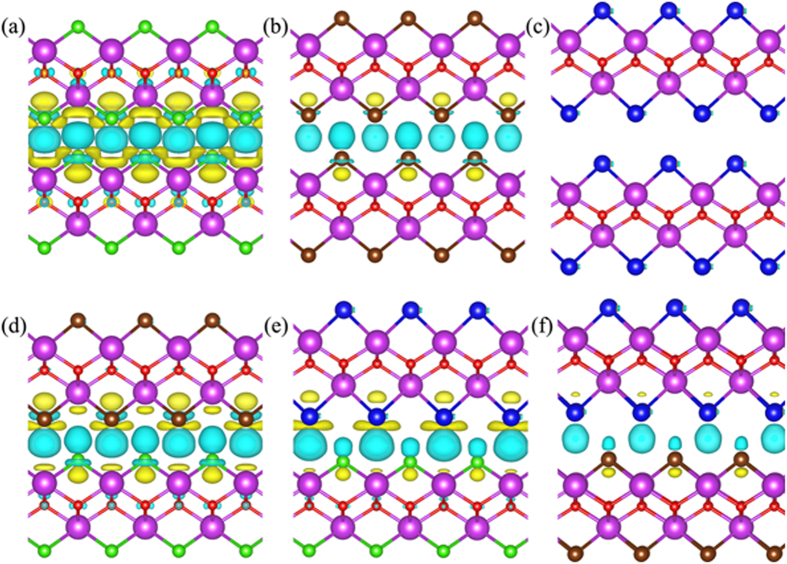
The corresponding charge density difference of the (**a**) BiOCl/BiOCl, (**b**) BiOBr/BiOBr, (**c**) BiOI/BiOI, and (**d**) BiOCl/BiOBr, (**e**) BiOCl/BiOI, (**f**) BiOBr/BiOI structures. The violet, red, green, brown, and blue balls represent Bi, O, Cl, Br, and I atoms, respectively. The yellow and blue-green isosurfaces correspond to the accumulation and depletion of electronic densities (the isovalue is 0.002 e/Å^3^).

To further analyze the basic physics of the interfacial interactions of a BiOX1/BiOX2 bilayer, we show the charge density distributions in the layer structure and charge density difference across the bilayer, with charge density difference defined as follows^34^:


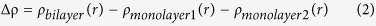


where *ρ*_*bilayer*_(*r*), *ρ*_*monolayer*1_(*r*) and *ρ*_*monolayer*2_(*r*) are the charge densities of the bilayer BiOX1/BiOX2, monolayer BiOX1, and BiOX2, respectively. The calculated results are summarized in Fig. 2(a,f). Briefly, bilayer coupling leads to the formation of interfacial dipole arising from charge redistribution centering between proximity pairs of Cl-Bi at the bilayer interface. The highest charge redistribution is found in the BiOCl/BiOCl structure (Fig. 2(a)), while the smallest charge redistribution is found in the BiOI/BiOI structure (Fig. 2(c)). This finding reflects the high electronegativity of Cl and relatively low electronegativity of I, the most critical atomic parameter which affects the differences in charge redistribution in the present case. By identifying the dipole nature of bilayer coupling in BiOX1/BiOX2, one can deduce a logical correlation of strong dipole with short interfacial bilayer separation. The bilayer separation data included in Table 1 indeed show such a correlation, with the following trend in bilayer separation: BiOCl/BiOCl < BiOCl/BiOBr < BiOBr/BiOBr < BiOCl/BiOI < BiOBr/BiOI < BiOI/BiOI.

Following the confirmation of strong bilayer coupling and thermodynamical stability of BiOX1/BiOX2, we analyze their applications-oriented properties, particularly water-splitting properties and other functional features derived from their basic electronic structures.

**Figure 3:**
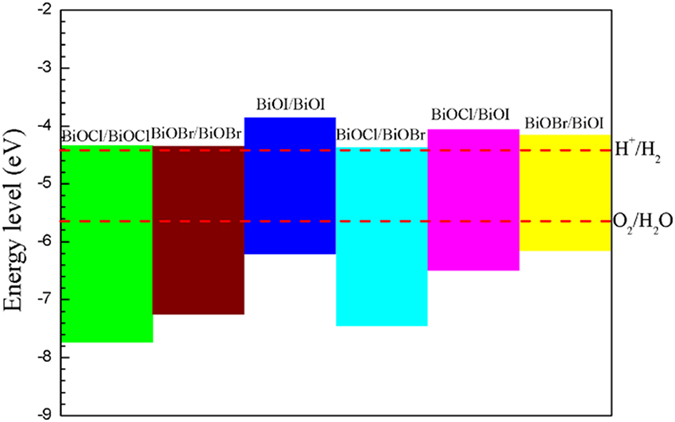
Comparison between the band edge positions of BiOCl/BiOCl, BiOBr/BiOBr, BiOI/BiOI, BiOCl/BiOBr, BiOCl/BiOI and BiOBr/BiOI structures related to the vacuum level calculated with the HSE06 functional.

The most critical requirement of electronic structural properties in assessing the candidacy of photocatalyst for water splitting (comprising a half reaction of H^+^/H_2_ and another half reaction of O_2_/H_2_O) is the alignment of the conduction band minimum (CBM) of the photocatalyst above the standard reduction potential for H^+^/H_2_ (at −4.44 eV below the vacuum level), and the valence band maximum (VBM) of the photocatalyst below the oxidation potential for O_2_/H_2_O (at −5.67 eV). The calculated band alignment data for all combinations of BiOX1/BiOX2 are summarized in Fig. 3. Clearly, all BiOX1/BiOX2 bilayers miraculously satisfy the above band alignment requirements for water splitting. Among these combinations, the bilayers of BiOI/BiOI, BiOCl/BiOI and BiOBr/BiOI have both attractive over-potentials for water splitting, and band gaps and appropriate bandgaps favoring visible light-absorption (BiOBr/BiOBr: 2.88 eV, BiOI/BiOI: 2.35 eV, BiOCl/BiOI: 2.43 eV, and BiOBr/BiOI: 1.85 eV).

**Figure 4:**
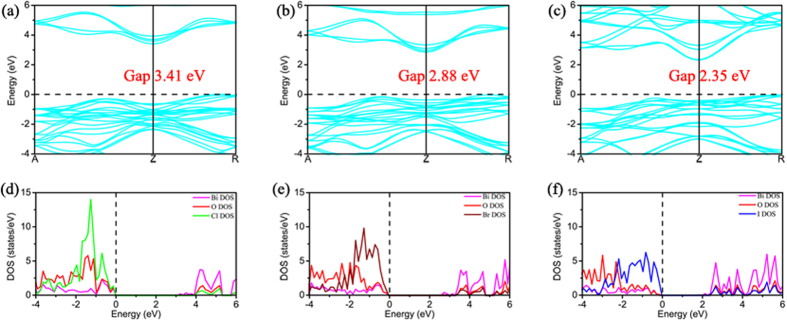
The band structures of the (**a**) BiOCl/BiOCl, (**b**) BiOBr/BiOBr, and (**c**) BiOI/BiOI structures. The corresponding partial density of states (DOS) of the (**d**) BiOCl/BiOCl, (**e**) BiOBr/BiOBr, and (**f**) BiOI/BiOI structures. The violet, red, green, brown, and blue lines represent the partial DOS of Bi, O, Cl, Br, and I atoms, respectively.

**Figure 5:**
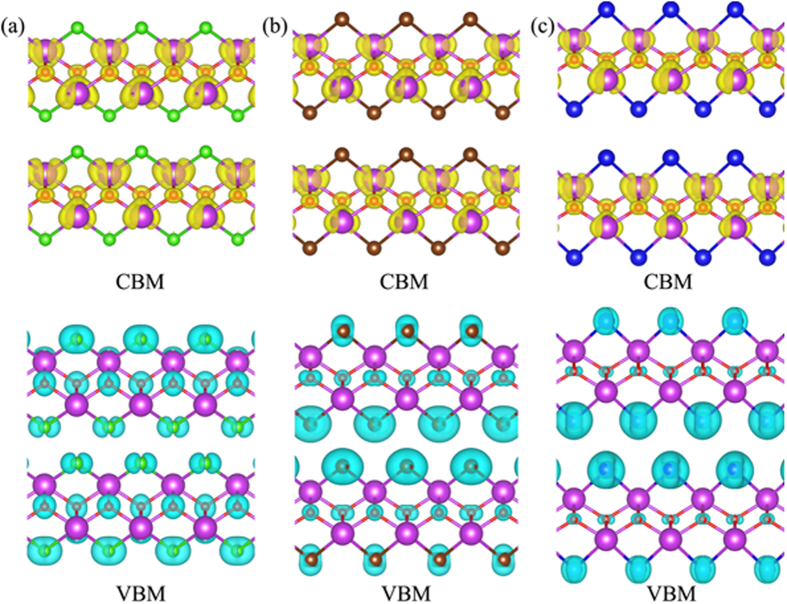
The band decomposed charge densities of (**a**) BiOCl/BiOCl, (**b**) BiOBr/BiOBr, and (**c**) BiOI/BiOI bilayer structures. The conduction band minimum (CBM) and valence band maximum (VBM) are shown on the upper and low panel in blue-green color (the isovalue is 0.03 e/Å^3^). The violet, red, green, brown, and blue balls represent Bi, O, Cl, Br, and I atoms, respectively.

To examine whether some of the BiOX1/BiOX2 bilayers are effective in separating photo-generated electron-hole pairs, with supplying electrons to the conduction band for facilitating the reduction-half-reaction of H^+^/H_2_ and supplying holes to the valence band for facilitating the oxidation-half-reaction of O_2_/H_2_O, we probe the detailed band structures, density of states (DOS), and charge density partitions into localized DOS near band-edges for all BiOX1/BiOX2 bilayers. First, the band structures of BiOCl/BiOCl, BiOBr/BiOBr, and BiOI/BiOI, as shown in the Fig. 4(a–c), reveal that the respective minimum band separations are 3.41, 2.88 and 2.35 eV, and all in the form of indirect bandgap. The partial DOS of BiOCl/BiOCl, BiOBr/BiOBr, and BiOI/BiOI show that DOS of the CBM of the bilayer are mainly contributed by Bi atoms, while DOS of the VBM of the bilayer are mainly contributed by X and O atoms. This special DOS nature can also be articulated by showing the partial charge densities corresponding to those DOS at CBM and VBM (Fig. 5(a–c)). For the case of BiOCl/BiOCl, occupied-electron states at VBM are mainly localized at the sites of the Cl atoms, particularly of the Cl atoms away from the bilayer interface, with the O atoms also contributing significantly to such occupied-electron states. Superimposing to this picture is that unoccupied states ready to accept electrons are localized at the sites of the Bi atoms, particularly for those Bi atoms near the bilayer interface. As such, photo-excitation manifests itself by mainly pumping electrons from the Cl atoms locating away from the interface, with some electrons from the O atoms, all in those states near VBM, to the empty CBM states belonging to the Bi atoms, particularly to those Bi atoms locating near the bilayer interface. Due to the homogeneous nature of the case of BiOCl/BiOCl, photo-excitation leads to pumping electrons from the VBM states of one layer constituent to the empty CBM states of the same layer constituent. In other words, there is no strong spatial separation of photo-generated electron-hole pairs. In addition, photo-generated holes in VBM are preferentially produced on the top/bottom surfaces of the bilayer, but photo-generated electrons in CBM are preferentially produced below the top/bottom surfaces of the bilayer near the bilayer interface. As such, while the O_2_/H_2_O half-reaction is well facilitated with easy supply of holes in VBM on the top/bottom surfaces of the bilayer, the H^+^/H_2_ half reaction is less well supported by the supply of electrons in CBM because most of the electron suppliers are located below the top/bottom surfaces.

The cases of BiOBr/BiOBr and BiOI/BiOI are similar to the case of BiOCl/BiOCl, except that the VBM contributions are dominated by the Br and I atoms locating near the bilayer interface, with less and less contributions from O atoms. Similar to the case of BiOCl/BiOCl, the empty CBM states are dominated by the Bi atoms locating near the bilayer interface. As such, photo-generated holes in VBM and electrons in CBM are all mainly produced near the bilayer interface, and the condition of spatial separation of photo-generated electron-hole pairs is even worse than the case of BiOCl/BiOCl. More specifically, both the O_2_/H_2_O half-reaction and the H^+^/H_2_ half reaction are not well facilitated, as the VBM holes and CBM electrons are generated away from the top/bottom surfaces of the bilayer.

**Figure 6:**
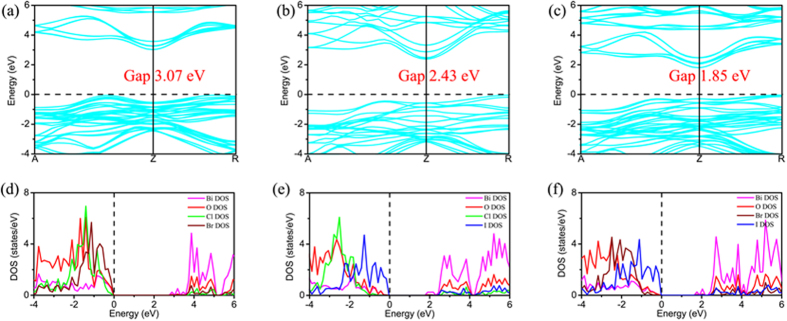
The band structures of the (**a**) BiOCl/BiOBr, (**b**) BiOCl/BiOI, and (**c**) BiOBr/BiOI heterostructures. The corresponding partial density of states (DOS) of the (**d**) BiOCl/BiOBr, (**e**) BiOCl/BiOI, and (**f**) BiOBr/BiOI structures. The violet, red, green, brown, and blue lines represent the partial DOS of Bi, O, Cl, Br, and I atoms, respectively.

**Figure 7:**
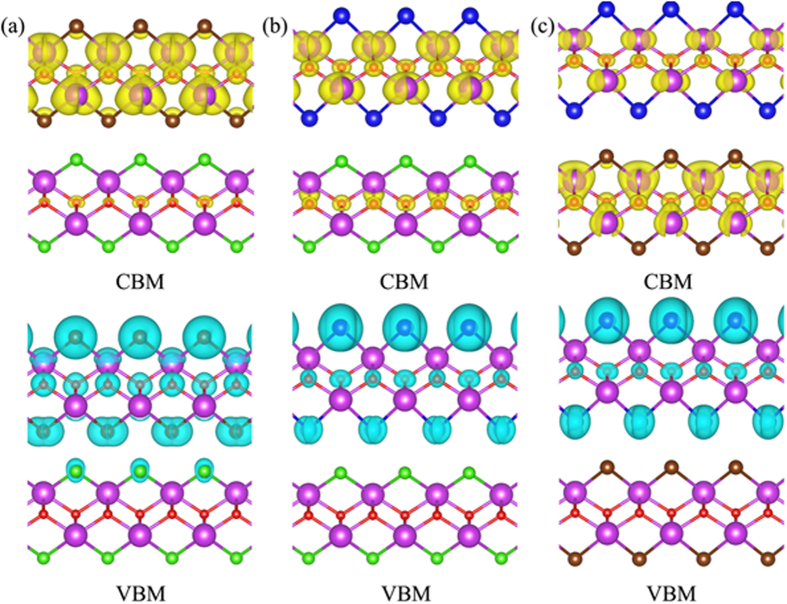
The band decomposed charge densities of the (**a**) BiOCl/BiOBr, (**b**) BiOCl/BiOI, and (**c**) BiOBr/BiOI heterostructures. The conduction band minimum (CBM) and valence band maximum (VBM) are shown on upper and low panel in blue-green color as well (the isovalue is 0.03 e/Å^3^). The violet, red, green, brown, and blue balls represent Bi, O, Cl, Br, and I atoms, respectively.

The heterogeneous coupling of two BiOX layer constituents having different X lifts some of the uncooperative spatial distributions of photo-generated VBM holes and CBM electrons towards water-splitting. Take the case of BiOBr/BiOI as an exemplar: Figs 6(c) and 7(c) show that photo-generated VBM holes are mainly produced at the I atom-sites on the surface of the BiOI layer constituent, with the photo-generated CBM electrons mainly produced at the Bi atom-sites of the BiOBr layer constituent. As such, the O_2_/H_2_O half-reaction is well facilitated on the surface of the BiOI layer constituent of the BiOBr/BiOI bilayer, with the H^+^/H_2_ half reaction being facilitated with the CBM electrons supplied by the Bi atoms of the BiOBr layer constituent. In this reaction process, H^+^ readily reaches the CBM electron supplier below the surface of the BiOBr layer constituent via diffusion. The reaction product of H_2_ can also be released easily via diffusion and gas-evolution.

In comparison to the case of BiOBr/BiOI, the case of BiOCl/BiOI is less favorable for water-splitting because BiOCl/BiOI has a bandgap of 2.43 eV, wider than the bandgap of BiOBr/BiOI at 1.85 eV. More importantly, Figs 6(b) and 7(b) show that while photo-generated VBM holes are mainly produced at the I atom-sites on the surface of the BiOI layer constituent, the photo-generated CBM electrons in this case of BiOCl/BiOI are also mainly produced at the Bi atom-sites of the BiOI layer constituent. As such, while the O_2_/H_2_O half-reaction is well facilitated on the surface of the BiOI layer constituent of the BiOCl/BiOI bilayer, the H^+^/H_2_ half reaction is also facilitated with the CBM electrons supplied by the Bi atoms of the BiOI layer constituent. The mechanism of spatial separation of photo-generated electron-hole pairs in the case of BiOBr/BiOI is absent in the case of BiOCl/BiOI.

Similarly, Figs 6(a) and 7(b) also show that the mechanism of spatial separation of photo-generated electron-hole pairs in the case of BiOBr/BiOI is absent in the case of BiOCl/BiOBr. Making the case of BiOCl/BiOBr worse is the fact that the bandgap of BiOCl/BiOBr is 3.07 eV, large enough to become insensitive to visible light.

**Figure 8:**
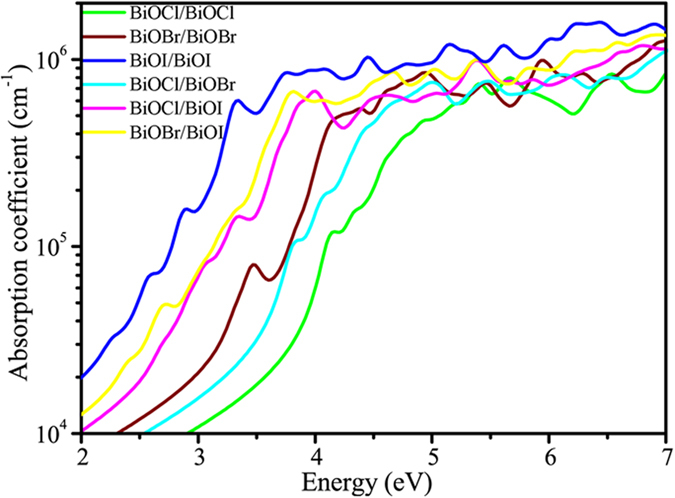
The calculated optical absorption spectra of BiOX1/BiOX2 bilayers. The green, brown, blue, blue-green, violet, and yellow lines represent the optical absorption spectra of the BiOCl/BiOCl, BiOBr/BiOBr, BiOI/BiOI, BiOCl/BiOBr, BiOCl/BiOI and BiOBr/BiOI structures, respectively.

To further examine the critical issue of photocatalytic efficiency, we probe and compare the optical optical absorption characertistics of all BiOX1/BiOX2 bilayers, with results shown in Fig. 8. Although many reports in the literature have advocated BiOI monolayer and BiOBr/BiOI bilayer as good photocatalysts for water-splitting, our calculated results reveal the common deficiency of all BiOX and BiOX1/BiOX2 bilayers being semiconductors having indirect bandgap. From the Fig. 8, we can see that the BiOI/BiOI and BiOBr/BiOI bilayers have an obvious adsorption peak at the visible light region. The first adsorption peak of BiOI/BiOI and BiOBr/BiOI locate at 2.91 eV and 2.71 eV, respectively. Although the BiOBr/BiOI structure have the lowest band gap, the optical adsorption property is not the best because of the optical adsorption property is not only depend on the band gap of the indirect semiconductors. The optical adsorption property depend on the transition probability between the VBM and CBM. However, the BiOBr/BiOI structure also have appropriate optical adsorption (10^5^ cm^−1^) at the visible light region. In future research, it is particularly useful to find novel means of band-structure engineering to further raise the visible optical absorption of BiOX1/BiOX2 bilayers, particularly the BiOBr/BiOI bilayer.

Finally, we note that although spatial separation of photo-generated electron-hole pairs of several heterogeneous bilayers have been speculated^35^,^36^,^37^,^38^,^39^, the spatial separation of these local electron/hole states are typically valid only at the CBM/VBM edges but these bilayers generally do not possess high optical absorptivity in such VBM-CBM band-edge excitation. In contrast, in the case of the BiOBr/BiOI bilayer, the domination of local DOS belong to the I atoms of the BiOI layer is valid from the VMB edge to about 1eV below the edge, and the domination of the local DOS belong to the Bi atoms of the BiOBr layer is also valid from the CMB edge to about 4eV above the edge. As such, the intriguing band-structural property facilitating spatial separation of photo-generated electron-hole pairs for water splitting persists in a relative wide range of photo-excitation spectrum from 2 to 5eV in the case of BiOBr/BiOI. This outstanding property has never been revealed in all known bilayer systems; as such, we advocate extensive follow-up studies of BiOBr/BiOI and its derivatives as efficient photocatalysts for water splitting.

## Discussions

Based on HSE06 hybrid density functional calculations, the electronic structures and optical properties of homo-bilayer bismuth oxyhalides (BiOX_1_/BiOX_2_, X_1_ = X_2_ = Cl, Br, I) and their hetero-bilayer counterparts (X_1_ ≠ X_2_) are studied. Among all BiOX_1_/BiOX_2_, BiOBr/BiOI bilayer is the best photocatalyst for water splitting because it has the smallest bandgap of 1.85 eV, and only this bilayer can facilitate spatial separation of photo-generated electron-hole pairs. In brief, the electron supply to the conduction band minimum with localized states residing mainly at the Bi atom-sites of the BiOBr layer constituents. Meanwhile, the holes in the valence band maximum of the BiOBr/BiOI bilayer are mainly produced at the I atom-sites on the surface of the BiOI layer constituent.

## Methods

The first-principles calculations were performed by the Vienna Ab Initio Simulation Package (VASP)^40^,^41^. Projector augmented-wave (PAW) pseudopotentials were used to account for electron-ion interactions. The generalized gradient approximation (GGA) with the PBE functional was used to treat the exchange-correlation interactions between electrons. A vacuum region larger than 20 Å perpendicular to the sheets (along the **c** axis) was applied to avoid the interactions between layers caused by the periodic boundary condition. The energy cutoff was set to 500 eV and a 11 × 11 × 1 Monkhorst-Pack scheme was used to sample the Brillouin zone. The full geometry optimizations were carried out with the convergence thresholds of 10^−6^ eV and 1 × 10^−3^ eV/Å for total energy and ionic force, respectively. For probing vdW interactions, the DFT-D3 approach was used^42^. To obtain accuracy electronic structures and bandgaps, the hybrid Heyd-Scuseria-Ernzerhof (HSE06)^43^,^44^ functionals calculations including spin-orbit coupling (SOC) effect were adopted.

## Additional Information

**How to cite this article**: Tang, Z.-K. *et al*. Spatial separation of photo-generated electron-hole pairs in BiOBr/BiOI bilayer to facilitate water splitting. *Sci. Rep.* **6**, 32764; doi: 10.1038/srep32764 (2016).

## Figures and Tables

**Table 1:** The detailed lattice constants, interlayer distances, formation energies, band edge positions, and band gaps of bilayer BiOX_1_/BiOX_2_,where a, D, E_f_, E_VB_, E_CB_, and Gap are the relaxed lattice parameter, optimized interlayer distance, formation energies, band edge positions of valence band maximum (VBM), band edge positions of conduction band minimum (CBM), and band gaps respectively.

Structure	a (Å)	D (Å)	E_f_ (eV)	E_VB_ (eV)	E_CB_ (eV)	Gap (eV)
BiOCl/BiOCl	3.885	2.179	−0.38	−7.77	−4.36	3.41
BiOBr/BiOBr	3.924	2.554	−0.32	−7.24	−4.36	2.88
BiOI/BiOI	4.000	3.012	−0.32	−6.25	−3.90	2.35
BiOCl/BiOBr	3.903	2.378	−0.33	−7.47	−4.40	3.07
BiOCl/BiOI	3.939	2.634	−0.27	−6.56	−4.13	2.43
BiOBr/BiOI	3.960	2.798	−0.29	−6.09	−4.24	1.85

## References

[b1] Fujishima A. & Honda K. Electrochemical Photolysis of Water at a Semiconductor Electrode. Nature 238, 37–38, 10.1038/238037a0 (1972). 12635268

[b2] Sun L. . Enhanced Visible-Light Photocatalytic Activity of BiOI/BiOCl Heterojunctions: Key Role of Crystal Facet Combination. ACS Catal. 5, 3540–3551, 10.1021/cs501631n (2015).

[b3] Wang H. . Semiconductor heterojunction photocatalysts: design, construction, and photocatalytic performances. Chem. Soc. Rev. 43, 5234–5244, 10.1039/C4CS00126E (2014). 24841176

[b4] Moniz S. J. A., Shevlin S. A., Martin D. J., Guo Z.-X. & Tang J. Visible-light driven heterojunction photocatalysts for water splitting - a critical review. Energ. Environ. Sci. 8, 731–759, 10.1039/C4EE03271C (2015).

[b5] Luntz A. C., Voss J. & Reuter K. Interfacial Challenges in Solid-State Li Ion Batteries. The journal of physical chemistry letters 6, 4599–4604, 10.1021/acs.jpclett.5b02352 (2015). 26551954

[b6] Zhou W. . Synthesis of Few-Layer MoS2 Nanosheet-Coated TiO2 Nanobelt Heterostructures for Enhanced Photocatalytic Activities. Small 9, 140–147, 10.1002/smll.201201161 (2013). 23034984

[b7] Dutta A. . Oriented Attachments and Formation of Ring-on-Disk Heterostructure Au–Cu3P Photocatalysts. Chem. Mater. 28, 1872–1878, 10.1021/acs.chemmater.6b00050 (2016).

[b8] Liang N. . Novel Bi2S3/Bi2O2CO3 heterojunction photocatalysts with enhanced visible light responsive activity and wastewater treatment. J. Mater. Chem. A 2, 4208–4216, 10.1039/C3TA13931J (2014).

[b9] Long M. . Efficient visible light photocatalytic heterostructure of nonstoichiometric bismuth oxyiodide and iodine intercalated Bi2O2CO3. Appl. Catal. B: Environ. 184, 20–27, 10.1016/j.apcatb.2015.11.025 (2016).

[b10] Lee C.-H. . Atomically thin p–n junctions with van der Waals heterointerfaces. Nat. Nano. 9, 676, 10.1038/nnano.2014.150 (2014). 25108809

[b11] Bin O., Fanchao M. & Jun S. Energetics and kinetics of vacancies in monolayer graphene boron nitride heterostructures. 2D Mater. 1, 035007, 10.1088/2053-1583/1/3/035007(2014).

[b12] Xiaofeng Q., Yangyang W., Wenbin L., Jing L. & Ju L. Modelling of stacked 2D materials and devices. 2D Mater. 2, 032003 (2015).

[b13] Rahman M. Z., Kwong C. W., Davey K. & Qiao S. Z. 2D phosphorene as a water splitting photocatalyst: fundamentals to applications. Energy Environ. Sci. 9, 709–728, 10.1039/C5EE03732H (2016).

[b14] Xue J. . Scanning tunnelling microscopy and spectroscopy of ultra-flat graphene on hexagonal boron nitride. Nat. Mater. 10, 282–285, 10.1038/nmat2968 (2011). 21317900

[b15] Dean C. R. . Boron nitride substrates for high-quality graphene electronics. Nat. Nano. 5, 722–726, 10.1038/nnano.2010.172 (2010). 20729834

[b16] Yang W. . Epitaxial growth of single-domain graphene on hexagonal boron nitride. Nat. Mater. 12, 792–797, 10.1038/nmat3695 (2013). 23852399

[b17] Shim G. W. . Large-Area Single-Layer MoSe2 and Its van der Waals Heterostructures. ACS Nano 8, 6655, 10.1021/nn405685j (2014). 24987802

[b18] Ceballos F., Bellus M. Z., Chiu H.-Y. & Zhao H. Ultrafast Charge Separation and Indirect Exciton Formation in a MoS2–MoSe2 van der Waals Heterostructure. ACS Nano 8, 12717, 10.1021/nn505736z (2014). 25402669

[b19] Deng Y. . Black Phosphorus–Monolayer MoS2 van der Waals Heterojunction p–n Diode. ACS Nano 8, 8292–8299, 10.1021/nn5027388 (2014). 25019534

[b20] Kośmider K. & Fernández-Rossier J. Electronic properties of the MoS_2_-WS_2_ heterojunction. Phys. Rev. B 87, 10.1103/PhysRevB.87.075451 (2013).

[b21] Ramasubramaniam A., Naveh D. & Towe E. Tunable Band Gaps in Bilayer Graphene−BN Heterostructures. Nano Lett. 11, 1070, 10.1021/nl1039499(2011). 21275424

[b22] Terrones H., López-Urías F. & Terrones M. Novel hetero-layered materials with tunable direct band gaps by sandwiching different metal disulfides and diselenides. Sci. Rep. 3, 1549, 10.1038/srep01549 (2013). 23528957 PMC3607896

[b23] Kang J., Li J., Li S.-S., Xia J.-B. & Wang L.-W. Electronic Structural Moiré Pattern Effects on MoS2/MoSe2 2D Heterostructures. Nano Lett. 13, 5485, 10.1021/nl4030648 (2013). 24079953

[b24] Henle J., Simon P., Frenzel A., Scholz S. & Kaskel S. Nanosized BiOX (X = Cl, Br, I) Particles Synthesized in Reverse Microemulsions. Chem. Mater. 19, 366–373, 10.1021/cm061671k (2007).

[b25] Zhang X., Ai Z., Jia F. & Zhang L. Generalized One-Pot Synthesis, Characterization, and Photocatalytic Activity of Hierarchical BiOX (X = Cl, Br, I) Nanoplate Microspheres. J. Phys. Chem. C 112, 747–753, 10.1021/jp077471t (2008).

[b26] An H. . Photocatalytic properties of BiOX (X = Cl, Br, and I). Rare Metals 27, 243–250, 10.1016/S1001-0521(08)60123-0 (2008).

[b27] Chang X. . BiOX (X = Cl, Br, I) photocatalysts prepared using NaBiO3 as the Bi source: Characterization and catalytic performance. Catal. Commun. 11, 460–464, 10.1016/j.catcom.2009.11.023 (2010).

[b28] Cheng H., Huang B. & Dai Y. Engineering BiOX (X = Cl, Br, I) nanostructures for highly efficient photocatalytic applications. Nanoscale 6, 2009–2026, 10.1039/C3NR05529A (2014). 24430623

[b29] Zhang X. . The stabilities and electronic structures of single-layer bismuth oxyhalides for photocatalytic water splitting. Phys. Chem. Chem. Phys. 16, 25854–25861, 10.1039/C4CP03166K (2014). 25354143

[b30] Cao J., Xu B., Luo B., Lin H. & Chen S. Novel BiOI/BiOBr heterojunction photocatalysts with enhanced visible light photocatalytic properties. Catal. Commun. 13, 63–68, 10.1016/j.catcom.2011.06.019 (2011).

[b31] Pease R. S. Crystal Structure of Boron Nitride. Nature 165, 722–723 (1950). 15416803 10.1038/165722b0

[b32] Zhou S., Han J., Dai S., Sun J. & Srolovitz D. J. van der Waals bilayer energetics: Generalized stacking-fault energy of graphene, boron nitride, and graphene/boron nitride bilayers. Phys. Rev. B 92, 155438 (2015).

[b33] Baskin Y. & Meyer L. Lattice Constants of Graphite at Low Temperatures. Phys. Rev. 100, 544–544 (1955).

[b34] Tang Z.-K., Zhang Y.-N., Zhang D.-Y., Lau W.-M. & Liu L.-M. The stability and electronic properties of novel three-dimensional graphene-MoS2 hybrid structure. Sci. Rep. 4, 10.1038/srep07007 (2014). PMC4228343 25387832

[b35] Kośmider K. & Fernández-Rossier J. Electronic properties of the MoS2-WS2heterojunction. Phys. Rev. B 87, 10.1103/PhysRevB.87.075451 (2013).

[b36] Bernardi M., Palummo M. & Grossman J. C. Extraordinary sunlight absorption and one nanometer thick photovoltaics using two-dimensional monolayer materials. Nano Lett. 13, 3664–3670, 10.1021/nl401544y (2013). 23750910

[b37] Liao J., Sa B., Zhou J., Ahuja R. & Sun Z. Design of High-Efficiency Visible-Light Photocatalysts for Water Splitting: MoS2/AlN(GaN) Heterostructures. J. Phys. Chem. C 118, 17594–17599, 10.1021/jp5038014 (2014).

[b38] Debbichi L., Eriksson O. & Lebègue S. Electronic structure of two-dimensional transition metal dichalcogenide bilayers fromab initiotheory. Phys. Rev. B 89, 10.1103/PhysRevB.89.205311 (2014).

[b39] Wei X. L., Tang Z. K., Guo G. C., Ma S. & Liu L. M. Electronic and magnetism properties of two-dimensional stacked nickel hydroxides and nitrides. Sci. Rep. 5, 11656, 10.1038/srep11656 (2015). 26111476 PMC4481523

[b40] Kresse G. & Furthmüller J. Efficient iterative schemes for ab initio total-energy calculations using a plane-wave basis set. Phys. Rev. B 54, 11169–11186, 10.1103/PhysRevB.54.11169 (1996). 9984901

[b41] Kresse G. & Furthmüller J. Efficiency of ab-initio total energy calculations for metals and semiconductors using a plane-wave basis set. Comput. Mater. Sci. 6, 15–50, 10.1016/0927-0256(96)00008-0 (1996). 9984901

[b42] Grimme S., Antony J., Ehrlich S. & Krieg H. A consistent and accurate ab initio parametrization of density functional dispersion correction (DFT-D) for the 94 elements H-Pu. J. Chem. Phys. 132, 154104, 10.1063/1.3382344 (2010). 20423165

[b43] Heyd J., Scuseria G. E. & Ernzerhof M. Hybrid functionals based on a screened Coulomb potential. J. Chem. Phys. 118, 8207–8215, 10.1063/1.1564060 (2003).

[b44] Heyd J., Scuseria G. E. & Ernzerhof M. Erratum: “Hybrid functionals based on a screened Coulomb potential” [J. Chem. Phys.118, 8207 (2003)]. J. Chem. Phys. 124, 219906, 10.1063/1.2204597 (2006).

